# Rural-Urban Disparities in Hospital Services and Outcomes for Children With Medical Complexity

**DOI:** 10.1001/jamanetworkopen.2024.35187

**Published:** 2024-09-24

**Authors:** JoAnna K. Leyenaar, Seneca D. Freyleue, Mary Arakelyan, Andrew P. Schaefer, Erika L. Moen, Andrea M. Austin, David C. Goodman, A. James O’Malley

**Affiliations:** 1 Department of Pediatrics, Children’s Hospital at Dartmouth-Hitchcock Medical Center, Lebanon, New Hampshire; 2 The Dartmouth Institute for Health Policy & Clinical Practice, Geisel School of Medicine at Dartmouth College, Hanover, New Hampshire; 3 Department of Biomedical Data Science, Geisel School of Medicine at Dartmouth College, Hanover, New Hampshire

## Abstract

**Importance:**

Limited availability of inpatient pediatric services in rural regions has raised concerns about access, safety, and quality of hospital-based care for children. This may be particularly important for children with medical complexity (CMC).

**Objectives:**

To describe differences in the availability of pediatric services at acute care hospitals where rural- and urban-residing CMC presented for hospitalization; identify rural-urban disparities in health care quality and in-hospital mortality; and determine whether the availability of pediatric services at index hospitals or the experience of interfacility transfer modified rural-urban differences in outcomes.

**Design, Setting, and Participants:**

This retrospective cohort study examined all-payer claims data from Colorado, Massachusetts, and New Hampshire from 2012 to 2017. Analysis was conducted from May 2023 to July 2024. Participants included CMC younger than 18 years residing in these states and hospitalized during the study period.

**Exposures:**

Rural or urban residence was determined using Rural-Urban Commuting Area codes. Hospitals were categorized as children’s hospitals or general hospitals with comprehensive, limited, or no dedicated pediatric services using American Hospital Association survey data. Interfacility transfers between index and definitive care hospitals were identified using health care claims.

**Main Outcomes and Measures:**

In-hospital mortality, all-cause 30-day readmission, medical-surgical safety events, and surgical safety events were operationalized using Agency for Healthcare Research and Quality measure specifications.

**Results:**

Among 36 943 CMC who experienced 79 906 hospitalizations, 16 525 (44.7%) were female, 26 034 (70.5%) were Medicaid-insured, and 34 008 (92.1%) were urban-residing. Rural-residing CMC were 6.55 times more likely to present to hospitals without dedicated pediatric services (rate ratio [RR], 6.55 [95% CI, 5.86-7.33]) and 2.03 times more likely to present to hospitals without pediatric beds (RR, 2.03 [95% CI, 1.88-2.21]) than urban-residing CMC, with no significant differences in interfacility transfer rates. In unadjusted analysis, rural-residing CMC had a 44% increased risk of in-hospital mortality (RR, 1.44 [95% CI, 1.03-2.02]) with no significant differences in other outcomes. Adjusting for clinical characteristics, the difference in in-hospital mortality was no longer significant. Index hospital type was not a significant modifier of observed rural-urban outcomes, but interfacility transfer was a significant modifier of rural-urban differences in surgical safety events.

**Conclusions and Relevance:**

In this cohort study, rural-residing CMC were significantly more likely to present to hospitals without dedicated pediatric services. These findings suggest that efforts are justified to ensure that all hospital types are prepared to care for CMC.

## Key Points

**Question:**

Among children with medical complexity requiring hospitalization, how do hospitals’ pediatric services differ for children who are rural- and urban-residing, and are hospitals’ pediatric services a significant modifier of rural-urban disparities in health outcomes?

**Findings:**

In this cohort study of 36 943 children from 3 states, rural-residing children were 6 times as likely to present for care at hospitals without dedicated pediatric services and they experienced significantly greater in-hospital mortality risk. However, hospitals’ availability of pediatric services was not a significant modifier of the association between rurality and health outcomes.

**Meaning:**

These results suggest that efforts to support high-quality care for children with medical complexity across the spectrum of hospital types are needed.

## Teaser

This cohort study examines whether differences in the availability of pediatric services at acute care hospitals where rural- and urban-residing children with medical complexity presented for hospitalization were associated with rural-urban disparities in health care quality and in-hospital mortality.

## Introduction

Children with medical complexity (CMC) are a diverse group of children with chronic medical conditions, functional limitations, and specialized health care needs.^1^ Although their underlying health conditions and health care needs are heterogenous, together CMC comprise a population at increased risk of poor health care quality, frequent hospital admission, readmission, and adverse health outcomes.^2,3,4,5^ A recent analysis found that CMC experienced at least 10 times the odds of in-hospital mortality relative to children without medical complexity.^6^ Despite these increased risks, the influence of health system infrastructure on health outcomes in CMC is not well understood.

Rural residence may place CMC at particularly increased risk of adverse health outcomes given their specialized health care needs coupled with limited access to specialized pediatric services in most rural communities.^7,8,9^ Rural-residing children receive fewer ambulatory care services and have more emergency department (ED) visits and hospitalizations than their urban-residing peers.^10^ However, rural-urban differences in where CMC receive their hospital-based care have not been previously examined. Health care quality and outcomes may be influenced by the availability of pediatric services at the hospital where CMC initially present for care,^11^ as well as by the experience of interfacility transfer,^12^ which may result in treatment delays and fragmented care. While the closure of pediatric units, particularly in rural areas, has been the focus of recent study and substantial media attention, the extent to which rural-residing CMC continue to seek care at these hospitals has not been previously explored.^13^

To address these knowledge gaps, this study aimed to describe differences in the availability of pediatric services at acute care hospitals where rural- and urban-residing CMC requiring hospitalization received their care; identify rural-urban disparities in health care quality measures and in-hospital mortality; and determine whether these differences were moderated by the experience of interfacility transfer or availability of pediatric services at hospitals where CMC presented for care.

## Methods

### Study Design and Data Source

We conducted a retrospective cohort study of all-payer claims data (APCD) from Colorado (CO), New Hampshire (NH), and Massachusetts (MA), which included data from Medicaid and employee-sponsored commercial plans spanning a 5-year period (January 1, 2013, to December 31, 2017, for NH and MA; and October 1, 2012, to September 30, 2017, for CO). APCD analytic files included member files indicating the duration of enrollment in participating health care plans, as well as professional and facility claims files reflecting health care encounters incurred across settings and payers. Acquisition, storage, and analysis of the APCD datasets were guided by data use agreements with (1) the Center for Improving Value in Health Care in CO, (2) NH Comprehensive Health Care Information System and Department of Health and Human Services, and (3) MA Center for Health Information and Analysis. These states were selected from the relatively limited number of states that make APCD available for research to represent regions with varied rural-urban composition. The Dartmouth Health Committee for the Protection of Human Subjects approved this study, determining that the criteria for approval at 45 CFR 46.111 and 21 CFR 56.111 were satisfied as appropriate. Reporting follows the Strengthening the Reporting of Observational Studies in Epidemiology (STROBE) reporting guideline.^14^

### Study Population

We analyzed data representing children younger than 18 years who were residents of CO, NH, or MA (N = 2 497 589), excluding those with a zip code that could not be linked to a Rural-Urban Commuting Area^15^ (RUCA) code (n = 532). CMC were identified by applying the Complex Chronic Conditions Classification System and the Pediatric Medical Complexity Algorithm to the first 3 years of data, consistent with prior validation studies.^6,16,17^ Given past research showing that these 2 algorithms identify different populations of children, we applied the 2 algorithms concurrently to *International Classification of Diseases, Ninth Revision (ICD-9)* and *ICD-10* codes documented on health care claims from any setting using previously published code that required eligible diagnoses to be documented on more than 1 unique date.^4^ The study cohort was limited to CMC who met criteria for either algorithm and experienced 1 or more hospitalizations (observation or inpatient status) or in-hospital deaths at acute care hospitals on or after their CMC cohort entry date (the date they met CMC diagnostic criteria). While the cohort was limited to CMC residing in our study’s 3 states, all US hospitalizations were included (acknowledging that CMC may cross state lines for care). Birth hospitalizations were excluded, as were hospitalizations at chronic care, psychiatric, and rehabilitation hospitals, and hospitals that could not be linked to 2015 or 2016 American Hospital Association (AHA) survey data or the Centers for Medicare and Medicaid Services Provider of Services (POS) file.^18,19^

For each CMC we measured, at the time of cohort entry, rurality using residential zip codes linked to RUCA codes (with rural including large rural and small town/rural regions and urban including urban core and suburban regions),^15,20^ age in years, gender (female, male, other/unknown) and state of residence. We additionally assessed primary payer (any Medicaid or exclusively commercial payers), number of hospitalizations during the observation period, and total months of enrollment and/or claims following the CMC diagnosis date. Clinical characteristics included specific body systems with chronic disease diagnoses (eTable 1 in Supplement 1), a binary indicator of technology assistance,^16^ and an indicator of a progressive complex disease (eg, muscular dystrophy or cystic fibrosis).^17^ Co-occurring disability diagnoses were identified using the Children with Disabilities Algorithm, requiring eligible diagnoses on at least 2 separate dates.^21^ To ensure that these characteristics preceded outcome assessment, they were derived using data prior to the date of hospital presentation. Missing data were reported and included as a variable category in regression analyses. We categorized each hospitalization as elective or nonelective, and as mental, medical, or surgical. Mental health hospitalizations were defined as those with a primary diagnosis included in the Child and Adolescent Mental Health Disorder Classification System.^22^ To identify surgical hospitalizations during the *ICD-9* period, we used Agency for Healthcare Research and Quality (AHRQ) Clinical Classification System (CCS) procedure flags to identify procedures and then applied the Surgery Flags Software for Services and Procedures to identify the subset of surgical procedures. To identify surgical procedures during the *ICD-10* period, we similarly applied the ARHQ CCS procedure flags and classified procedures as surgical when at least 50% of procedures were surgical per the *ICD-9* surgical flags.^19,20,21,23,24,25^All other hospitalizations were categorized as medical.

### Hospital Characteristics

Acute care hospitals where CMC received care were categorized into 4 mutually exclusive groups using AHA survey data following a previously established, hierarchical approach: (1) freestanding children’s hospitals (FCH, defined as hospitals that “restrict admissions primarily to children”)^19^; (2) hospitals with comprehensive pediatric services (hospitals with a pediatric ED, neonatal intensive care unit [ICU], and pediatric ICU); (3) hospitals with limited pediatric services (hospitals with any but not all of: pediatric ED, neonatal ICU, pediatric ICU, or pediatric beds); and (4) hospitals with no dedicated pediatric services (hospitals lacking all of: pediatric ED, neonatal ICU, pediatric ICU, and pediatric beds).^23^ Hospitals missing an AHA survey response were characterized using the POS file, augmented with online searches of hospital websites for pediatric ED services.^18^ These datasets were also used to identify hospitals’ geographic location (rural vs urban), critical access hospital status, membership in the Council of Teaching Hospitals (teaching hospital status), medical school affiliation, and number of pediatric beds.

Hospitals where CMC presented for care, whether they received ED, observation, or inpatient care, were categorized as index hospitals. Hospitals where CMC completed their care, whether observation or inpatient status, were identified as *definitive care* settings. We identified interfacility transfers from index hospitals to definitive care hospitals, excluding transfers to psychiatric, chronic care, and rehabilitation hospitals. We evaluated length of stay (LOS) for each hospitalization, counting the number of hospital days across both the index and definitive care hospitals when applicable.

### Outcome Measures

We examined 4 outcomes, including in-hospital mortality and 3 nationally endorsed quality measures derived from AHRQ’s Pediatric Quality Measures Program. In-hospital mortality was determined using discharge disposition codes coupled with cessation of subsequent in-person health care claims. Medical-surgical safety events were evaluated as a composite measure of accidental puncture or laceration, iatrogenic pneumothorax, and/or central venous catheter-related blood stream infection in CMC hospitalized with medical or surgical indications (excluding hospitalizations with a primary mental health diagnosis).^24,25,26^ Because these are rare events, they were combined as a single binary-valued outcome variable to adhere to data use agreements which prohibit reporting events experienced by fewer than 11 individuals. Surgical safety events were similarly evaluated as a composite measure of postoperative respiratory failure, postoperative sepsis, and perioperative hemorrhage or hematoma in CMC hospitalized for surgical indications.^27,28,29^ Finally, we evaluated 30-day all-condition hospital readmission. Per AHRQ measure specification, hospitalizations with principal diagnoses of mental health or obstetrics-related conditions and readmissions for chemotherapy or other planned procedures were excluded.^30^ Outcomes were attributed to the hospitalization episode (index plus definitive care).

### Statistical Analysis

Sociodemographic and clinical characteristics of CMC meeting inclusion criteria were reported descriptively using frequencies and percentages or means and SDs. Frequencies and percentages were also calculated to characterize index hospital characteristics for CMC by state of residence, and to describe sociodemographic and clinical characteristics of CMC based on index hospital type. Characteristics of hospitalizations, including index hospital characteristics, receipt of interfacility transfer, and LOS were compared between rural- and urban-residing CMC using Poisson regression implemented with a log-link function. For estimation we used generalized estimating equations (GEEs) with an exchangeable correlation structure to account for statistical dependences among repeated observations for CMC with multiple hospitalizations.^31,32^ Similarly, we compared outcomes between rural- and urban-residing CMC using robust Poisson regression procedures. Initial models were unadjusted (model 1). To account for baseline demographic and clinical characteristics, we used inverse propensity score weighting that made the mean treatment effect size across our study sample the target of inference (model 2). Weights were calculated as the inverse of the probability of a CMC being urban- or rural-residing, based on a model including age, gender, state, body systems affected by complexity, progressive condition indicator, co-occurring disability, and technology dependence. Because these characteristics were potentially time-varying, they were reassessed for each hospitalization using data prior to index hospital presentation. Covariate balance was assessed with standardized differences, and covariates that remained unbalanced were also included as covariates in the outcome models (eTable 2 in Supplement 1). Finally, to evaluate potential modification of outcomes by rural-urban differences in the availability of pediatric services at index hospitals and in interfacility transfer status, we developed a third set of models that extended model 2 by adding additional covariates including index hospital type, interfacility transfer, and interaction terms between rurality and index hospital type and rurality and interfacility transfer when statistically significant (model 3). The same propensity score weights were used for models 2 and 3, and for these models we also accounted for correlation at the hospital level using GEEs. Analyses were conducted using SAS version 9.4 (SAS Institute) from May 2023 to July 2024. Statistical testing was 2-sided, and *P* < .05 was considered statistically significant.

## Results

### Study Population

A total of 36 943 CMC experienced 79 906 hospitalizations; 20 328 (55.0%) were male and 16 525 (44.7%) were female; 26 034 (70.5%) were insured by Medicaid; and 34 008 (92.1%) were urban-residing and 2935 (7.9%) were rural-residing (Table 1). A relatively larger proportion of rural-residing CMC were insured by Medicaid (2213 [75.4%] vs 23 821 [70.0%] urban-residing CMC) and had chronic disease diagnoses affecting a single body system (1966 [67.0%] vs 20 059 [59.0%] urban-residing CMC). CMC experienced a mean (SD) of 2.2 (2.6) hospitalizations during the study period.

**Table 1.** Baseline Characteristics of Rural- and Urban-Residing CMC Who Had at Least 1 Inpatient Stay During the Study Period

Characteristics	CMC, No. (%)		
	Total (n = 36 943)	Urban-residing (n = 34 008)	Rural-residing (n = 2935)
Age, y^a^			
<2	12 400 (33.6)	11 321 (33.3)	1079 (36.8)
2-5	7184 (19.4)	6654 (19.6)	530 (18.1)
6-11	8245 (22.3)	7631 (22.4)	614 (20.9)
12-15	7164 (19.4)	6638 (19.5)	526 (17.9)
16-17	1950 (5.3)	1764 (5.2)	186 (6.3)
Gender			
Male	20 328 (55.0)	18 729 (55.1)	1599 (54.5)
Female	16 525 (44.7)	15 205 (44.7)	1320 (45)
Missing or unknown	90 (0.2)	74 (0.2)	16 (0.5)
Primary payor			
Any Medicaid	26 034 (70.5)	23 821 (70.0)	2213 (75.4)
Commercial only	10 909 (29.5)	10 187 (30.0)	722 (24.6)
No. of body systems with chronic condition diagnoses (%)			
1	22 025 (59.6)	20 059 (59.0)	1966 (67.0)
2	9811 (26.6)	9204 (27.1)	607 (20.7)
3	2999 (8.1)	2782 (8.2)	217 (7.4)
≥4	2108 (5.7)	1963 (5.8)	145 (4.9)
Progressive condition	8656 (23.4)	7978 (23.5)	678 (23.1)
Co-occurring disability	18 162 (49.2)	16 693 (49.1)	1469 (50.1)
Technology assistance	2256 (6.1)	2060 (6.1)	196 (6.7)
State			
Colorado	13 967 (37.8)	12 387 (36.4)	1580 (53.8)
Massachusetts	20 436 (55.3)	20 104 (59.1)	332 (11.3)
New Hampshire	2540 (6.9)	1517 (4.5)	1023 (34.9)
No. of hospitalization episodes, mean (SD)	2.2 (2.6)	2.2 (2.7)	2 (2.4)
Observation, mean (SD), mo	29.9 (15.6)	30.1 (15.6)	27.2 (14.9)

Abbreviation: CMC, children with medical complexity.

^a^ Age at time of first hospitalization following entry into the cohort.

### Patterns of Hospitalization

Overall, 32 761 hospitalizations (41.0%) began at FCH, 21 756 (27.2%) at hospitals with comprehensive pediatric services, 23 054 (28.6%) at hospitals with limited pediatric services, and 2335 (2.9%) at hospitals without dedicated pediatric services (Table 2). Similar proportions of rural- and urban-residing CMC received their initial hospital care at hospitals with limited pediatric services. However, 809 rural-residing CMC (13.5%) presented to index hospitals without dedicated pediatric services, a rate that was 6.55 times that observed in urban-residing CMC (rate ratio [RR], 6.55 [95% CI, 5.86-7.33]). Rural-residing CMC were also 23% less likely to present for care at hospitals affiliated with medical schools (RR, 0.77 [95% CI, 0.75-0.80]) and twice as likely to present to hospitals without pediatric beds (RR, 2.03 [95% CI, 1.88-2.21]).

**Table 2.** Characteristics of Hospitalizations and RRs Experienced by Rural-Residing CMC Compared With Urban-Residing CMC

Characteristics	CMC, No. (%)			Rural vs urban CMC, RR (95% CI)^a^
	Total (n = 79 906)	Urban (n = 73 927)	Rural (n = 5979)	
Index hospital characteristics^b^				
Availability of pediatric services				
Freestanding children’s hospitals (n = 39)	32 761 (41)	30 976 (41.9)	1785 (29.9)	0.71 (0.67-0.76)
Comprehensive pediatric services (n = 78)	21 756 (27.2)	20 067 (27.1)	1689 (28.2)	1.04 (0.96-1.13)
Limited pediatric services (n = 161)	23 054 (28.6)	21 358 (28.9)	1696 (28.4)	0.98 (0.92-1.05)
No dedicated pediatric services (n = 84)	2335 (2.9)	1526 (2.1)	809 (13.5)	6.55 (5.86-7.33)
Critical access hospital (n = 52)	634 (0.8)	102 (0.1)	532 (8.9)	64.5 (48.7-85.4)
Rurally located hospital (n = 75)	3825 (4.8)	1013 (1.4)	2812 (47.0)	34.3 (30.6-38.5)
Teaching hospital (n = 88)	44 294 (55.4)	42 145 (57)	2149 (35.9)	0.63 (0.59-0.67)
Hospital with medical school affiliation (n = 208)	68 944 (86.3)	64 878 (87.8)	4066 (68.0)	0.77 (0.75-0.8)
No. of pediatric beds^c^				
None	9708 (12.4)	8336 (11.5)	1372 (23.3)	2.03 (1.88-2.21)
1-20	17872 (22.7)	16 692 (23)	1180 (20.1)	0.87 (0.80-0.95)
21-200	30 484 (38.8)	27 743 (38.2)	2741 (46.6)	1.22 (1.16-1.28)
>200	20 498 (26.1)	19 910 (27.4)	588 (10.0)	0.37 (0.31-0.43)
Receipt of interfacility transfer	17 530 (21.9)	16 198 (21.9)	1332 (22.3)	1.02 (0.95-1.08)
Length of stay, mean (SD), d	6.1 (20.6)	6 (21)	6.4 (13.8)	1.06 (0.99-1.13)
Principal diagnosis				
Mental health	5997 (7.5)	5690 (7.7)	307 (5.1)	0.67 (0.57-0.78)
Medical	64 602 (80.8)	59 571 (80.6)	5031 (84.1)	1.04 (1.03-1.06)
Surgical	9307 (11.6)	8666 (11.7)	641 (10.7)	0.91 (0.83-1.01)
Elective hospitalization	11 835 (14.8)	11 076 (15.0)	759 (12.7)	0.85 (0.77-0.93)

Abbreviations: CMC, children with medical complexity; RR, rate ratio.

^a^ RRs are derived from Poisson regressions with robust error variances and patient-level clustering to account for multiple hospitalizations for a given CMC during the study period.

^b^ Numbers in parentheses represent the number of hospitals included in this analysis.

^c^ Number of pediatric beds missing info for 1246 hospitalizations in urban-residing CMC and 98 hospitalizations in rural-residing CMC.

Analysis of state-level variation in index hospital characteristics showed similar patterns across each state (eTable 3 in Supplement 1), but relatively more hospitalizations began at FCH in MA and CO compared with NH. The largest proportion of hospitalizations at hospitals with limited pediatric services occurred in CO; within CO we also observed the largest magnitude of rural-urban difference in the proportion of CMC presenting for care at hospitals without dedicated pediatric services: 1.6% (408 of 24 927) urban-residing CMC in CO presented at these hospitals, compared with 18.7% (569 of 3035) of rural-residing CMC. We also observed differences in sociodemographic and clinical characteristics across index hospital types (eTable 4 in Supplement 1). Children who presented for initial hospital care at hospitals with limited or no pediatric services were more frequently insured by Medicaid and adolescents, and less likely to have co-occurring disabilities or be assisted by technology.

In total, 1332 rural-residing CMC (22.3%) and 16 198 urban-residing CMC (21.9%) experienced an interfacility transfer, a difference that was not statistically significant (RR, 1.02 [95% CI, 0.95-1.08]). The overall LOS did not differ significantly between rural- and urban-residing CMC. Mental health diagnoses were significantly less common (RR, 0.67 [95% CI, 0.57-0.78]), and medical hospitalizations significantly more common (RR, 1.04 [95% CI, 1.03-1.06]) among rural-residing CMC. Elective hospitalizations were also significantly less common among rural-residing CMC (RR, 0.85 [95% CI, 0.77-0.93]).

Alluvial plots of flow across index and definitive care hospital types (Figure) showed that, among rural-residing CMC, 53.4% (n = 432) of hospitalizations beginning at hospitals without dedicated pediatric services resulted in interfacility transfers; the proportion was slightly higher (56.4% [n = 860]) for urban-residing CMC who initially presented to these hospitals. In total, 7.1% (n = 424) of hospitalizations for rural-residing CMC were completed at hospitals without dedicated pediatric services, representing 46.4% (n = 377) of hospitalizations that began at these hospitals. In contrast, 1.5% (n = 1134) of hospitalizations for urban-residing CMC were completed at hospitals without dedicated pediatric services, representing 43.6% (n = 666) of hospitalizations that began at these hospitals. eTable 5 in Supplement 1 provides frequencies and percentages for all hospitalizations.

**Figure.**
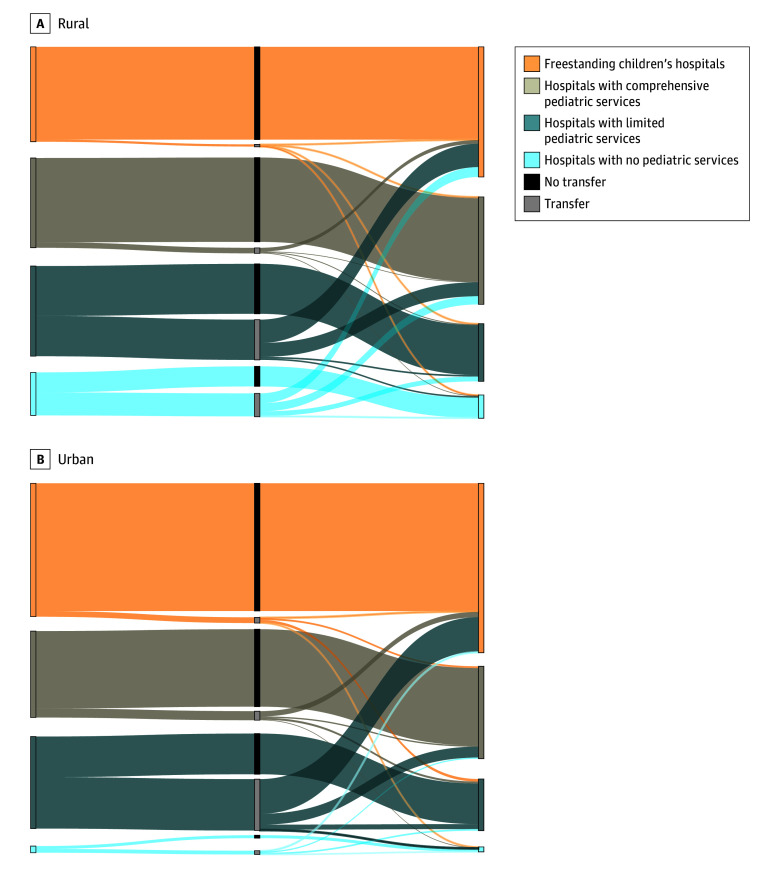
Availability of Pediatric Resources at Index Hospitals and Definitive Care Hospitals and Experience of Interfacility Transfer Among Urban- and Rural-Residing Children With Medical Complexity These alluvial plots show availability of pediatric resources at index hospitals and definitive care hospitals and experience of interfacility transfer among urban- and rural-residing children with medical complexity. All n values less than 11 were set to 0 to abide by the data use agreement that applied Centers for Medicare & Medicaid Services suppression rules. Freestanding children’s hospitals primarily serve children; comprehensive pediatric services include hospitals with a pediatric emergency department, neonatal intensive care unit (NICU), and pediatric intensive care unit (PICU); hospitals with limited pediatric services include hospitals with any but not all of the following: pediatric emergency department, NICU, PICU, or pediatric beds; hospitals with no pediatric services reported none of these services.

### Health Outcomes

In unadjusted analysis, there were no significant differences between rural- and urban-residing CMC in the AHRQ quality measures, but rural-residing CMC had a 44% higher in-hospital mortality rate (RR, 1.44 [95% CI, 1.03-2.02) (Table 3). When adjusting for baseline clinical and demographic characteristics, the rural-urban difference in mortality was no longer statistically significant (RR, 0.90 [95% CI, 0.58-1.38]).

**Table 3.** Quality and Safety Outcomes in Rural- vs Urban-Residing CMC at the Hospitalization-Level

	Hospitalizations in urban-residing CMC	Hospitalizations in rural-residing CMC	RR (95% CI)		
			Model 1: unadjusted	Model 2: adjusted for clinical characteristics^a^	Model 3: adjusted for clinical and hospitalization characteristics^b^
Medical-surgical safety events, No. (events per 10 000)^c^	441 (64.6)	32 (56.4)	0.87 (0.61-1.25)	1.13 (0.56-2.27)	1.19 (0.57-2.48)
Surgical safety events, No. (events per 10 000)^d^	366 (422.3)	27 (421.2)	1.00 (0.68-1.46)	0.71 (0.32-1.58)	0.84 (0.46-1.53)
30-d All-cause readmission, No. (%)^e^	10 255 (15.0)	903 (15.9)	1.06 (1.00-1.13)	0.90 (0.70-1.17)	0.91 (0.70-1.19)
In-hospital mortality, No. (events per 10 000)	325 (44.0)	38 (63.6)	1.44 (1.03-2.02)	0.9 (0.58-1.38)	0.86 (0.54-1.37)

Abbreviations: CMC, children with medical complexity; RR, rate ratio.

^a^ Weighted by the inverse probability of urban- or rural-residence based on a model using age, gender, state, body systems affected by complexity, progressive condition indicator, co-occurring disability, and technology dependence as factors, and also including indicators of cardiovascular, gastrointestinal, and/or hematological chronic disease diagnosis as separate covariates given imbalance in these characteristics and accounting for hospital-level clustering using generalized estimating equations.

^b^ Includes model 2 covariates as well as index hospital type and interfacility transfer status; the surgical safety event model also included the interaction between interfacility transfer status and rurality.

^c^ Medical-surgical safety events include accidental puncture or laceration, iatrogenic pneumothorax, and central venous catheter-related bloodstream infection. Based on 68 237 surgical and/or medical hospitalizations in urban-residing CMC and 5672 hospitalizations in rural-residing CMC.

^d^ Surgical safety events include postoperative respiratory failure, postoperative sepsis, and perioperative hemorrhage or hematoma. Based on 8666 surgical hospitalizations in urban-residing CMC and 641 hospitalizations in rural-residing CMC.

^e^ Based on 68 723 hospitalizations of urban-residing CMC and 5671 hospitalizations of rural-residing CMC.

Effect modification analyses found that index hospital type was not a significant modifier of rural-urban differences in health outcomes (eTable 6 in Supplement 1). Analyses of effect modification by interfacility transfer status are shown in eTable 7 in Supplement 1. The only significant interaction was observed for surgical safety events. Relative to urban-residing CMC who were not transferred, rural-residing CMC who were not transferred were significantly less likely to experience surgical safety events (RR, 0.44 [95% CI, 0.22-0.85]) while both urban- and rural-residing CMC who were transferred experienced significantly increased rates of this outcome. Overall, the adjusted RR of surgical safety events for rural-residing CMC compared with urban-residing CMC was 3.72 times greater in the presence of an interfacility transfer (RR, [95% CI, 1.37-10.09]).

## Discussion

This cohort study found that more than 30% of both urban- and rural-residing CMC requiring hospitalization initially presented for care at hospitals with limited or no pediatric services. Rural-residing CMC were 6.55 times as likely as their urban-residing peers to present to hospitals without dedicated pediatric services, and approximately half of these children (46.6%) completed their full course of care at these facilities. Pediatric service availability at index hospitals was not a significant modifier of differences in health outcomes between rural- and urban-residing CMC. However, interfacility transfer was a significant modifier of the risk of surgical safety events.

National analyses indicate that nonbirth pediatric hospitalizations declined by more than 25% from 2009 to 2019 while the relative proportion of hospitalizations experienced by CMC increased by more than 45% during this period.^33^ Analysis of AHA survey data during a similar period found that less than half of approximately 4400 surveyed hospitals had dedicated pediatric units, representing a 20% decrease from 2008 to 2018.^13^ This decrease was disproportionately experienced by rural hospitals. These findings have led to the conclusion that children presenting at these hospitals must be transferred to other hospitals for definitive care.^34^ While numerous studies have demonstrated increasing regionalization of pediatric care,^9,35,36^ the results of this analysis provide additional context: there were no significant differences in rates of interfacility transfer between rural- and urban-residing CMC, and more than half of rural-residing children who presented to hospitals with limited or no dedicated pediatric resources completed their full course of inpatient care in these settings. Although urban-residing CMC were less likely to present to hospitals without dedicated pediatric services, more than 40% of all children who presented to these hospitals similarly remained there for definitive care. Further research is needed to understand factors contributing to these findings, including the roles of parental preferences, clinician recommendations, and market forces such as local and regional pediatric bed availability and insurance coverage.

The increased mortality rate experienced by rural-residing CMC in this study is consistent with the known increased risk of childhood death in rural-residing children in the US.^37,38^ Following adjustment for demographic and clinical characteristics, the rural-urban difference in mortality was no longer statistically significant, suggesting that rural-residing CMC presented to hospitals with increased baseline risk. Although the availability of pediatric services at index hospitals was not a significant moderator of rural-urban differences in mortality or other outcomes in this analysis, several prior studies have shown that improved readiness of hospitals to provide pediatric care is associated with decreased mortality for children following trauma and with critical illnesses.^39,40^ While the pediatric readiness of hospitals included in this study is unknown, the increased mortality risk experienced by rural-residing CMC provides further evidence to support health policy and resource investment to ensure that all hospitals have the capacity to provide necessary care for CMC.

Our finding that the rural-urban RR of surgical safety events was 3.7 times higher in hospitalizations that involved a transfer than in those that did not illustrates the importance of the interfacility transfer process on health outcomes. It is possible that interfacility transfer may delay access to surgical services and thereby increase risk of adverse outcomes, particularly for rural-residing children who need to travel longer distances to reach pediatric surgical centers.^41,42^ Alternatively, surgical services performed at hospitals with lower pediatric volumes may be associated with increased adverse outcomes.^43,44,45^ Finally, it is possible that interfacility transfer status served as a marker of otherwise unmeasured clinical severity. Although the current study was not designed to evaluate the etiology of observed differences, our findings speak to the importance of ensuring high-quality interfacility transfer processes. Timely and appropriate interfacility transfers may be supported by individualized care plans for CMC that are accessible to clinicians across care settings, telehealth services to support hospitals with limited pediatric resources, and regional guidelines developed collaboratively by sending and receiving hospitals.^46,47,48,49^

### Limitations

Limitations of this study include the relative lack of clinical detail within APCD to characterize, with greater specificity, the quality of care provided during hospitalization, or clinical or nonclinical factors (eg, travel time) associated with observed outcomes. Relatedly, there are few validated or nationally endorsed all-condition quality measures for children that can be operationalized using health care claims data. Even with our large sample size, we were unable to report specific AHRQ medical and surgical Pediatric Quality Indicators, but instead created composites to abide by our data use agreements. The 3 states included in this study were selected to reflect varied rural-urban geographies, but the study population may not be nationally representative. We relied on AHA and POS files to determine hospital characteristics, which may have been associated with some misclassification. We also relied on previously established algorithms to identify CMC from claims data; these algorithms do not account for patient- or parent-reported functional impairment or other dimensions of medical complexity, but have been shown to identify children at substantially increased risk of in-hospital mortality and specialized health care needs.^6^ Importantly, the study period preceded COVID-19 and the numerous hospital closures and other health system changes that have occurred since COVID-19 onset.^50,51^ Given additional closures of pediatric units since 2017, the proportion of CMC presenting to hospitals without dedicated pediatric services may be underestimated.

## Conclusions

This cohort study found that a substantial fraction of CMC requiring hospitalization initially presented for care at hospitals without dedicated pediatric services. Rural-residing CMC experienced significantly higher in-hospital mortality, but this increased mortality risk did not persist following adjustment for demographic and clinical characteristics. Although the availability of comprehensive pediatric services at index hospitals did not appear to moderate rural-urban differences in health outcomes, interfacility transfer was a significant moderator of differences in the risk of adverse surgical outcomes. These results suggest that inclusion of diverse hospital types in research and clinical program supporting CMC is needed to support high quality care for this population.

## Notes

Supplement 1. **eTable 1.** Unified Categories of Body Systems Impacted by Chronic Disease Based on Two Open-Source Algorithms **eTable 2.** Demographic and Clinical Characteristics of Urban- and Rural-Residing Children With Medical Complexity at the Time of Hospitalization, Unweighted and Inverse Probability Weighted to Make the Average Treatment Effect Across Our Study Sample the Target of Inference **eTable 3.** Index Hospital Characteristics of Hospitalizations Among Children With Medical Complexity by State of Residence **eTable 4.** Characteristics of Children With Medical Complexity (CMC) at the Time of Hospitalization by Availability of Pediatric Resources at the Index Hospital **eTable 5.** Index and Definitive Hospital Types and Inter-Facility Transfer Status for Hospitalizations Among Rural- and Urban-Residing Children **eTable 6.** Modification of the Effect of Rural Residence on Healthcare Quality and Safety by Index Hospital Type (Free-Standing Children’s Hospitals/Comprehensive Pediatric Services Versus Limited/No Pediatric Services) **eTable 7.** Modification of the Effect of Rural Residence on Healthcare Quality and Safety by Inter-Hospital Transfer Status

Supplement 2.
Data Sharing Statement
