# Supplementary material for: Rural-Urban Disparities in Hospital Services and Outcomes for Children With Medical Complexity
Source: JAMA Netw Open. 2024 Sep 24;7(9):e2435187. doi: 10.1001/jamanetworkopen.2024.35187 (PMC11423179; doi:10.1001/jamanetworkopen.2024.35187)
Supplement: Supplement 1. — eTable 1. Unified Categories of Body Systems Impacted by Chronic Disease Based on Two Open-Source Algorithms eTable 2. Demographic and Clinical Characteristics of Urban- and Rural-Residing Children With Medical Complexity at the Time of Hospitalization, Unweighted and Inverse Probability Weighted to Make the Average Treatment Effect Across Our Study Sample the Target of Inference eTable 3. Index Hospital Characteristics of Hospitalizations Among Children With Medical Complexity by State of Residence eTable 4. Characteristics of Children With Medical Complexity (CMC) at the Time of Hospitalization by Availability of Pediatric Resources at the Index Hospital eTable 5. Index and Definitive Hospital Types and Inter-Facility Transfer Status for Hospitalizations Among Rural- and Urban-Residing Children eTable 6. Modification of the Effect of Rural Residence on Healthcare Quality and Safety by Index Hospital Type (Free-Standing Children’s Hospitals/Comprehensive Pediatric Services Versus Limited/No Pediatric Services) eTable 7. Modification of the Effect of Rural Residence on Healthcare Quality and Safety by Inter-Hospital Transfer Status [file jamanetwopen-e2435187-s001.pdf]

Supplemental Online Content

Leyenaar JK, Freyleue SD, Arakelyan M, et al. Rural-urban disparities in hospital services and outcomes for children with medical complexity. *JAMA Netw Open.* 2024;7(9):e2435187. doi:10.1001/jamanetworkopen.2024.35187

eTable 1. Unified categories of body systems impacted by chronic disease based on two open-source algorithms.

eTable 2. Demographic and clinical characteristics of urban- and rural-residing children with medical complexity at the time of hospitalization, unweighted and inverse probability weighted to make the average treatment effect across our study sample the target of inference.

eTable 3. Index hospital characteristics of hospitalizations among children with medical complexity by state of residence.

eTable 4. Characteristics of children with medical complexity (CMC) at the time of hospitalization by availability of pediatric resources at the index hospital

eTable 5. Index and definitive hospital types and inter-facility transfer status for hospitalizations among rural- and urban-residing children

eTable 6. Modification of the effect of rural residence on healthcare quality and safety by index hospital type (free-standing children's hospitals/comprehensive pediatric services versus limited/no pediatric services).

eTable 7. Modification of the effect of rural residence on healthcare quality and safety by inter-hospital transfer status.

This supplemental material has been provided by the authors to give readers additional information about their work.

© 2024 Leyenaar JK et al. *JAMA Network Open.*

eTable 1. Unified categories of body systems impacted by chronic disease based on two open-source algorithms.

Pediatric Medical Complexity Algorithms	Complex Chronic Condition Classification System	Unified Category
Progressive condition	-	Progressive
Malignancy	Malignancy	Malignancy
Cardiac condition	Cardiovascular	Cardiovascular
Craniofacial condition	-	Craniofacial
Dermatological condition	-	Dermatological
Endocrinological condition	-	Endocrinological
Gastrointestinal condition	Gastrointestinal	Gastrointestinal
Genetic condition	Congenital/genetic	Congenital/genetic
Genitourinary condition	-	Genitourinary
Hematological condition	Hematologic or immunologic	Hematologic or immunologic
Immunological condition		
Mental health condition	-	Mental health
Metabolic condition	Metabolic	Metabolic
Musculoskeletal condition	Neuromuscular	Neuromuscular
Neurological condition		
Ophthalmological condition	-	Ophthalmological
Otologic condition	-	Ear, nose, and throat
Otolaryngolical condition	-	
Pulmonary-respiratory condition	Respiratory	Respiratory
Renal condition	Renal	Renal
-	Neonatal	Neonatal
-	Transplantation	Transplantation
-	Technology dependence	Technology dependence

The Pediatric Medical Complexity Algorithm and Complex Chronic Conditions Classification System identify children and adolescents with medical complexity when applied to administrative claims data. Both algorithms were applied concurrently according to a previously published method.[4] Because the body systems identified by each algorithm are similar but not exactly the same, they were combined as shown above. Each child was assigned one or more "unified category."

© 2024 Leyenaar JK et al. *JAMA Network Open.*

eTable 2. Demographic and clinical characteristics of urban- and rural-residing children with medical complexity at the time of hospitalization, unweighted and inverse probability weighted to make the average treatment effect across our study sample the target of inference.[a]

	Unweighted			Weighted		
**Variable**	**Urban (n=73,927) n (%)**	**Rural (n=5,979) n (%)**	**St. Diff[b]**	**Urban %**	**Rural %**	**St. Diff[b]**
**Age**						
<2yrs	21,148 (28.6)	1,862 (31.1)	-0.055	28.8	28.6	0.003
2-5yrs	17,062 (23.1)	1,360 (22.7)	0.008	23.1	22.6	0.011
6-11yrs	16,949 (22.9)	1,306 (21.8)	0.026	22.9	23.1	-0.006
12-15yrs	14,205 (19.2)	1,024 (17.1)	0.054	19.1	20.4	-0.034
16-17 yrs	4,563 (6.2)	427 (7.1)	-0.039	6.2	5.3	0.039
**Gender**						
Female	33,394 (45.2)	2,633 (44.2)	-0.018	45.1	45.5	0.009
Male	40,408 (54.7)	3,323 (55.6)	0.016	54.8	54.3	0.018
Unknown/Other	125 (0.2)	23 (0.4)	0.041	0.2	0.2	-0.001
**Co-occurring disability**	44,203 (59.8)	3,486 (58.3)	0.03	59.6	60.7	-0.023
**Body system impacted by complexity [c]**						
Craniofacial condition	1,041 (1.4)	126 (2.1)	0.053	1.5	1.7	0.022
Cardiovascular condition	19,762 (26.7)	1,515 (25.3)	-0.032	26.6	22.2	***-0.101***
Dermatological condition	483 (0.7)	46 (0.8)	0.014	0.7	0.5	-0.018
Endocrinological condition	5,296 (7.2)	430 (7.2)	0.001	7.2	7.4	0.010
ENT condition	642 (0.9)	57 (1.0)	0.009	0.9	0.7	-0.017
Genetic condition	16,862 (22.8)	1,218 (20.4)	-0.059	22.6	25.8	0.077
Genitourinary condition	4,180 (5.7)	311 (5.2)	-0.02	5.7	4.8	-0.035
Gastrointestinal condition	16,765 (22.7)	1,296 (21.7)	-0.024	22.6	27.3	***0.113***
Hematological condition	10,027 (13.6)	582 (9.7)	***-0.12***	13.3	9.2	***-0.126***
Malignancy	6,981 (9.4)	635 (10.6)	0.039	9.5	8.3	-0.043
Metabolic condition	9,408 (12.7)	712 (11.9)	-0.025	12.6	10.0	-0.08
Neonatal condition	6,638 (9.0)	557 (9.3)	0.012	9.0	10.7	0.059
Neuromuscular condition	26,683 (36.1)	1,976 (33)	-0.064	35.8	38.6	0.058
Ophthalmological condition	5,625 (7.6)	253 (4.2)	***-0.143***	7.3	7.6	0.011
Progressive condition	32,103 (43.4)	2,433 (40.7)	-0.055	43.2	43.5	0.006
Renal condition	6,141 (8.3)	484 (8.1)	-0.008	8.3	8.2	-0.002
Respiratory condition	24,213 (32.8)	1,591 (26.6)	***-0.135***	32.3	33.4	0.026
Technology dependence	18325 (24.8)	1521 (25.4)	-0.015	24.8	26.5	-0.04
Transplant	2018 (2.7)	124 (2.1)	-0.043	2.7	2.2	-0.027
**State**						
Colorado	24927 (33.7)	3035 (50.8)	***-0.35***	35.0	34.5	0.01
Massachusetts	45786 (61.9)	683 (11.4)	***1.231***	58.1	58.5	-0.01
New Hampshire	3214 (4.3)	2261 (37.8)	***-0.9***	6.9	6.9	-0.001

[a] Statistical models were weighted by the inverse probability of urban- or rural-residence based on age, gender, co-occurring disability, state, body systems impacted by complexity, progressive condition indicator, and technology dependence as predictors, and also included indicators of cardiovascular, gastrointestinal, and/or hematological conditions as separate covariates given imbalance in these characteristics. [b] Statistically significant differences based on standardized differences <-0.1 or >0.1 are shown in bold.
[c] Each child is assigned one or more body systems impacted by complexity.

© 2024 Leyenaar JK et al. *JAMA Network Open.*

eTable 3. Index hospital characteristics of hospitalizations among children with medical complexity by state of residence.

	Colorado			Massachusetts			New Hampshire		
	Total n=27,962	Urban n=24,927	Rural n=3,035	Total n=46,469	Urban n=45,786	Rural n=683	Total n=5,475	Urban n=3,214	Rural n=2,261
**Index Hospital characteristics**									
Availability of pediatric services:									
Freestanding children's hospitals	11,601 (42.5%)	10,411 (41.8%)	1,190 (39.2%)	19,836 (42.7%)	19,606 (42.8%)	230 (33.7%)	1,324 (24.2%)	959 (29.8%)	365 (16.1%)
Comprehensive pediatric services	5,587 (20.0%)	5,210 (20.9%)	377 (12.4%)	13,610 (29.3%)	13,401 (29.3%)	209 (30.6%)	2,559 (46.7%)	1,456 (45.3%)	1,103 (48.8%)
Limited pediatric services	9,797 (35.0%)	8,898 (35.7%)	899 (29.6%)	11,900 (25.6%)	11,700 (25.6%)	200 (1.7%)	1,357 (24.8%)	760 (23.6%)	597 (26.4%)
No dedicated pediatric services	977 (3.5%)	408 (1.6%)	569 (18.7%)	1,123 (2.4%)	1,079 (2.4%)	44 (6.4%)	235 (4.3%)	39 (1.2%)	196 (8.7%)
Critical access hospital	293 (1.0%)	52 (0.2%)	241 (7.9%)	99 (0.2%)	22 (0.0%)	77 (11.3%)	242 (4.4%)	28 (0.9%)	214 (9.5%)
Rurally-located hospital	1,259 (4.5%)	304 (1.2%)	955 (31.5%)	233 (0.5%)	82 (0.2%)	151 (22.1%)	2,333 (42.6%)	627 (19.5%)	1,706 (75.5%)
Teaching hospital	5,697 (20.4%)	5,437 (21.8%)	260 (8.6%)	35,434 (76.3%)	34,953 (76.3%)	481 (70.4%)	3,163 (57.8%)	1,755 (54.6%)	1,408 (62.3%)
Hospital with medical school affiliation	21,808 (78.0%)	20,066 (80.5%)	1,742 (57.4%)	43,178 (92.9%)	42,667 (93.2%)	511 (74.8%)	3,958 (72.3%)	2,145 (66.7%)	1,813 (80.2%)

© 2024 Leyenaar JK et al. *JAMA Network Open.*

eTable 4. Characteristics of children with medical complexity (CMC) at the time of hospitalization by availability of pediatric resources at the index hospital

	Freestanding Children's Hospitals (n=32,761)	Hospitals with comprehensive pediatric services (n=21,756)	Hospitals with limited pediatric services (n=23,054)	Hospitals without dedicated pediatric services (n=2,335)
**Age, n (%)**				
<2yrs	9,192 (28.1%)	6,711 (30.8%)	6,638 (28.8%)	469 (20.1%)
2-5yrs	7,721 (23.6%)	5,120 (23.5%)	5,135 (22.3%)	446 (19.1%)
6-11yrs	8,232 (25.1%)	4,900 (22.5%)	4,615 (20.0%)	508 (21.8%)
12-15yrs	6,106 (18.6%)	3,739 (17.2%)	4,761 (20.7%)	623 (26.7%)
16-17yrs	1,510 (4.6%)	1,286 (5.9%)	1,905 (8.3%)	289 (12.4%)
**Gender, n (%)** [1]				
Male	18,005 (55.0%)	11,898 (54.7%)	12,567 (54.5%)	1,261 (54.0%)
Female	14,756 (45.0%)	9,858 (45.3%)	10,487 (45.5%)	1,074 (46.0%)
**Primary Payor, n (%)**				
Any Medicaid	21,496 (65.6%)	16,556 (76.1%)	17,818 (77.3%)	1,837 (78.7%)
Commercial Only	11,265 (34.4%)	5,200 (23.9%)	5,236 (22.7%)	498 (21.3%)
**Number of Body Systems with Chronic Condition Diagnoses, n (%)**				
1	11,379 (34.7%)	8,190 (37.6%)	9,161 (39.7%)	818 (35.0%)
2	7,377 (22.5%)	5,769 (26.5%)	6,754 (29.3%)	854 (36.6%)
3	4,409 (13.5%)	2,861 (13.2%)	2,833 (12.3%)	328 (14.0%)
4+	9,596 (29.3%)	4,936 (22.7%)	4,306 (18.7%)	335 (14.3%)
**Progressive condition, n (%)**	16,070 (49.1%)	9,284 (42.7%)	8,378 (36.3%)	804 (34.4%)
**Co-occurring disability, n (%)**	21,289 (65%)	12,500 (57.5%)	12,752 (55.3%)	1,148 (49.2%)
**Technology assistance, n (%)**	9,562 (29.2%)	4,942 (22.7%)	4,950 (21.5%)	392 (16.8%)

[1] Gender is other or unknown for 148 hospitalizations. To comply with data use agreement cell suppression rules, they have been combined with female.

© 2024 Leyenaar JK et al. *JAMA Network Open.*

eTable 5. Index and definitive hospital types and inter-facility transfer status for hospitalizations among rural- and urban-residing children

	Hospitalizations by rural-residing children (n=5,979)	Hospitalizations by urban-residing children (n=73,927)
**Index hospital type, n (%)**		
Freestanding children's hospitals	1,785 (29.9%)	30,976 (41.9%)
No transfer	*1,745 (97.8%)*	*29,664 (95.8%)*
Transfer	*40 (2.2%)*	*1,312 (4.2%)*
Hospitals with comprehensive pediatric services	1,689 (28.2%)	20,067 (27.1%)
No transfer	*1,586 (93.9%)*	*17,969 (89.5%)*
Transfer	*103 (6.1%)*	*2,098 (10.5%)*
Hospitals with limited pediatric services	1,696 (28.4%)	21,358 (28.9%)
No transfer	*939 (55.4%)*	*9,430 (44.2%)*
Transfer	*757 (44.6%)*	*11,928 (55.8%)*
Hospitals with no pediatric services	809 (13.5%)	1,526 (2.1%)
No transfer	*377 (46.6%)*	*666 (43.6%)*
Transfer	*432 (53.4%)*	*860 (56.4%)*
**Definitive hospital type, n (%)**		
Freestanding children's hospitals	2,448 (40.9%)	39,356 (53.2%)
Hospitals with comprehensive pediatric services	2,023 (33.8%)	21,452 (29.0%)
Hospitals with limited pediatric services	1,084 (18.1%)	11,985 (16.2%)
Hospitals with no pediatric services	424 (7.1%)	1,134 (1.5%)

© 2024 Leyenaar JK et al. *JAMA Network Open.*

eTable 6. Modification of the effect of rural residence on healthcare quality and safety by index hospital type (freestanding children's hospitals/comprehensive pediatric services versus limited/no pediatric services).

Medical/surgical safety events [a]					
	Urban		Rural		Medical/surgical safety events (rural vs. urban), RR (95% CI)
	N (per 10,000)	RR (95% CI)	N (per 10,000)	RR (95% CI)	
Comprehensive	342 (70.3)	1.0 (reference)	19 (56.3)	1.19 (0.57, 2.48)	1.19 (0.57, 2.48)
Limited/no services	99 (50.6)	0.43 (0.24, 0.78)	13 (56.5)	0.52 (0.28, 0.95)	
**Surgical safety events [b]**					
	Urban		Rural		Surgical safety events (rural vs. urban), RR (95% CI)
	N (per 10,000)	RR (95% CI)	N (per 10,000)	RR (95% CI)	
Comprehensive	307 (406.2)	1.0 (reference)	16 (304.8)	0.84 (0.46, 1.53)	0.84 (0.46, 1.53)
Limited/no services	59 (532.5)	0.73 (0.40, 1.32)	11 (948.3)	0.61 (0.27, 1.41)	
**All-cause 30 day Readmissions [c]**					
	Urban		Rural		Readmissions (rural vs. urban), RR (95% CI)
	N (%)	RR (95% CI)	N (%)	RR (95% CI)	
Comprehensive	7,721 (15.8)	1.0 (reference)	600 (17.8)	0.91 (0.70, 1.19)	0.91 (0.70, 1.19)
Limited/no services	2,534 (13.0)	0.88 (0.69, 1.13)	303 (13.2)	0.81 (0.63, 1.02)	
**In-hospital Mortality**					
	Urban		Rural		In-hospital mortality (rural vs. urban), RR (95% CI)
	N (per 10,000)	RR (95% CI)	N (per 10,000)	RR (95% CI)	
Comprehensive	188 (36.8)	1.0 (reference)	19 (54.7)	0.86 (0.54, 1.37)	0.86 (0.54, 1.37)
Limited/no services	137 (59.9)	1.65 (0.62, 4.34)	19 (75.8)	1.42 (0.56, 3.59)	

Presented are: 1) relative risks (RR) for each stratum of rurality and index hospital type with urban-residing CMC initiating care at hospitals with comprehensive services (freestanding children's hospitals and comprehensive pediatric services) as the reference group; 2) RRs for rural versus urban comparisons within strata of index hospital type (right-most column); 3) RRs of the interaction terms (a multiplicative measure of effect modification) in footnotes. When statistically significant effect modification was not observed, the right-most column presents a single RR for the rural-urban comparisons that applies to all CMC regardless of index hospital type. Models weighted by the inverse probability of urban- or rural-residence based on a model using age, gender, state, body systems impacted by complexity, progressive condition indicator, co-occurring disability, and technology dependence as predictors, and also including indicators of cardiovascular, gastrointestinal, and/or hematological chronic disease diagnosis as a separate covariate given imbalance in these characteristics. [a] Medical-surgical composite quality measure comprises accidental puncture or laceration, iatrogenic pneumothorax, and central venous catheter-related bloodstream infection. Based on 68,237 surgical and/or medical hospitalizations in urban-residing CMC and 5,672 hospitalizations in rural-residing CMC.

[b] Surgical composite quality measure comprises postoperative respiratory failure, postoperative sepsis, and perioperative hemorrhage or hematoma. Based on 8,666 surgical hospitalizations of urban-residing CMC and 641 hospitalizations of rural-residing CMC.

[c] Based on 68,723 hospitalizations of urban-residing CMC and 5,671 hospitalizations of rural-residing CMC.

© 2024 Leyenaar JK et al. *JAMA Network Open.*

eTable 7. Modification of the effect of rural residence on healthcare quality and safety by inter-hospital transfer status.

	Urban		Rural		
**Medical/surgical safety events [a]**					
	**Urban**		**Rural**		**Medical/surgical safety events (rural vs. urban), RR (95% CI)**
	N (per 10,000)	RR (95% CI)	N (per 10,000)	RR (95% CI)	
No transfer	337 (62.4)	1.0 (reference)	18 (40.7)	1.19 (0.57, 2.48)	1.19 (0.57, 2.48)
Transfer	104 (73.2)	0.50 (0.30, 0.83)	14 (112.1)	0.59 (0.23, 1.56)	
**Surgical safety events [b]**					
	**Urban**		**Rural**		**Surgical safety events (rural vs. urban), by transfer status RR (95% CI) [c]**
	N (per 10,000)	RR (95% CI)	N (per 10,000)	RR (95% CI)	
No transfer	287 (370.2)	1.0 (reference)	14 (255.5)	0.44 (0.22, 0.85)	0.44 (0.22, 0.85)
Transfer	79 (865.3)	2.88 (1.97, 4.21)	13 (1,397.8)	4.67 (2.01, 10.86)	1.62 (0.67, 3.90)
**All cause 30-day Readmissions [d]**					
	**Urban**		**Rural**		**Readmissions (rural vs. urban), RR (95% CI)**
	N (%)	RR (95% CI)	N (%)	RR (95% CI)	
No transfer	8,168 (15.1)	1.0 (reference)	720 (16.3)	0.91 (0.70, 1.19)	0.91 (0.70, 1.19)
Transfer	2,087 (14.7)	0.96 (0.77, 1.21)	183 (14.7)	0.88 (0.60, 1.29)	
**In-hospital Mortality**					
	**Urban**		**Rural [e]**		**In-hospital mortality (rural vs. urban), RR (95% CI)**
	N (per 10,000)	RR (95% CI)	N (per 10,000)	RR (95% CI)	
No transfer	234 (40.5)	1.0 (reference)	-	0.86 (0.54, 1.37)	0.86 (0.54, 1.37)
Transfer	91 (56.2)	1.11 (0.63, 1.97)	-	0.96 (0.44, 2.07)	

Presented are: 1) relative risks (RR) for each stratum of rurality and transfer status with urban-residing CMC not experiencing a transfer as the reference group; 2) RRs for rural versus urban comparisons within strata of transfer status (right-most column); 3) RRs of the interaction terms (a multiplicative measure of effect modification) in footnotes. When statistically significant effect modification was not observed, the right-most column presents a single RR for the rural-urban comparisons that applies to all CMC regardless of being transferred or not. Models weighted by the inverse probability of urban- or rural-residence based on a model using age, gender, state, body systems impacted by complexity, progressive condition indicator, co-occurring disability, and technology dependence as predictors, and also including indicators of cardiovascular, gastrointestinal, and/or hematological chronic disease diagnosis as a separate covariate given imbalance in these characteristics.

[a] Medical-surgical composite quality measure comprises accidental puncture or laceration, iatrogenic pneumothorax, and central venous catheter-related bloodstream infection. Based on 68,237 surgical and/or medical hospitalizations in urban-residing CMC and 5,672 hospitalizations in rural-residing CMC.
[b] Surgical composite quality measure comprises postoperative respiratory failure, postoperative sepsis, and perioperative hemorrhage or hematoma. Based on 8,666 surgical hospitalizations of urban-residing CMC and 641 hospitalizations of rural-residing CMC.
c Interaction RR: 3.72 (95%CI: 1.37, 10.09); p=0.001.
[d] Based on 68,723 hospitalizations of urban-residing CMC and 5,671 hospitalizations of rural-residing CMC.
[e] Cell sizes <11 and cells that would allow back-calculation suppressed per data use agreement.

© 2024 Leyenaar JK et al. *JAMA Network Open.*
