# Supplementary material for: Rural-Urban Disparities in Hospital Services and Outcomes for Children With Medical Complexity
Source: JAMA Netw Open. 2024 Sep 24;7(9):e2435187. doi: 10.1001/jamanetworkopen.2024.35187 (PMC11423179; doi:10.1001/jamanetworkopen.2024.35187)
Supplement: Supplement 2. — Data Sharing Statement [file jamanetwopen-e2435187-s002.pdf]

Data Sharing Statement

Leyenaar. Rural-Urban Disparities in Hospital Services and Outcomes for Children With Medical Complexity. *JAMA Netw Open*. Published September 24, 2024.
doi:10.1001/jamanetworkopen.2024.35187

Data
Data available: No

Additional Information
Explanation for why data not available: Our data use agreement prohibits sharing data at the patient level.
